# William Benjamin Carpenter and the Emerging Science of Heredity

**DOI:** 10.1007/s10739-019-09568-3

**Published:** 2019-06-26

**Authors:** John Lidwell-Durnin

**Affiliations:** Centre for the History of Science, Medicine, and Technology, History Faculty, Oxford University, 41-47 George Street, Oxford, UK

**Keywords:** William Benjamin Carpenter, Heredity, Inheritance of acquired characteristics, Lamarckism, Physiology, Unitarian

## Abstract

In the nineteenth century, farmers, doctors, and the wider public shared a family of questions and anxieties concerning heredity. Questions over whether injuries, mutilations, and bad habits could be transmitted to offspring had existed for centuries, but found renewed urgency in the popular and practical scientific press from the 1820s onwards. Sometimes referred to as “Lamarckism” or “the inheritance of acquired characteristics,” the potential for transmitting both desirable and disastrous traits to offspring was one of the most pressing scientific questions of the nineteenth century. As I argue in this paper, Carpenter’s religious commitments to abolition and the temperance movement shaped his understanding of heredity. But this also committed him to a body of evidence for the inheritance of acquired characteristics that was coming under criticism for being untrustworthy. Carpenter used his popular treatises on physiology to promote these older, familiar ideas about heredity because they provided vital means of arguing for the unity of mankind and the hereditary dangers of intemperance. While early nineteenth century physiology has been seen by some historians as a challenge to religious authority, given its potentially materialist accounts of the body and the actions of the soul, this paper demonstrates how the missionary and institutional activities of the Unitarian church were ideologically supported by Carpenter’s publications.

## Introduction

In 1839, the 26-year-old medical student William Benjamin Carpenter, seeking to establish a reputation as a leading physiologist, was fast at work on his *Principles of General and Comparative Physiology*. In a section titled “Hereditary Transmission of *acquired* peculiarities,” Carpenter included the observation that: “Cats deprived of their tails will often produce one or two tail-less kittens at each birth” (1839, p. 422). Providing a handful of similar observations, Carpenter upheld that the popular notion of the inheritance of acquired characteristics was true. Humans, like cats, dogs, and all other creatures, could potentially transmit injuries sustained in their lives to their offspring. It was an idea that had been rejected not only within physiology, but also within Carpenter’s own church. The Unitarian physiologist James Cowles Prichard had pronounced as early as 1813 that mutilations and injuries could not be transmitted. The falsehood of such an idea was, Prichard contended, “universally confirmed by experience” (1813, p. 196). “If a child was born without a foot,” Prichard observed, “it would not occur to any person to impute the want of the limb to any amputation with either of its parents might have undergone” (1813, p. 197). Why was Carpenter, a devoted student of Prichard’s works, so determined to defend a view of heredity that had been rejected by his predecessors as contrary to experience, ill-informed, and doubtful? The very question of what our experience confirms about heredity was at stake, and Carpenter, armed with a publishing contract from John Churchill in London, was beginning a lifelong campaign to contend that our experience *does* confirm the very sort of events that Prichard regarded as ridiculous.

The *Principles*, like everything Carpenter produced, presented leading and even radical scientific opinions of his time, but it was also woven with old and recycled threads borrowed from popular opinion, the agricultural press, and even Carpenter’s own religious commitments as the eldest son of a prominent Unitarian minister. Within the anecdotes and stories Carpenter included in his treatise lay scientific arguments for the religious causes of his day: the abolition movement, the temperance movement, and the education of the poor. A decade after Carpenter’s death, the scientist William Bateson would complain of the “indiscriminate accumulation of facts” that characterized this early period of investigation into heredity (1894, p. 7). Bateson charged that a “formless and vague” hope for discovery fueled the collection of such stories, but as I hope to demonstrate in this article, the purpose of the anecdotes and facts that Carpenter collected was to confirm what we already knew about the operations of nature. The content of our experience either supported the inheritance of acquired characteristics, or, as Prichard had maintained, it refuted the idea.

Carpenter wasn’t the only one at the time to believe that cats that lost their tails could breed tailless kittens. In 1841, the asylum surgeon W. A. F. Browne wrote that the inheritance of acquired characteristics was “part of the traditionary knowledge of the vulgar, and as one of those truths developed by the experience of the ages” (Browne 1841, p. 222). When we survey the proponents of the idea, this appeal to its universality reappears often. George Combe, in his *Constitution of Man* (1828), had also upheld the possibility of the inheritance of mutilations and injuries, borrowing his evidence from agricultural and medical periodicals (Jenkins 2015). But Carpenter’s books, unlike those of Combe, came to represent orthodox medical and scientific knowledge for many of his readers (Winter 1998, p. 41), and the discussion of cat tails wasn’t one that Carpenter was in any hurry to withdraw; it reappeared in the greatly revised edition of 1841 and was still present in revised edition of 1854, the edition that Charles Darwin used to familiarize himself with Karl Ernst von Baer’s embryology (Ospovat 1976). Due to the success and clout of his popular treatises on physiology, as well as his influential roles as registrar of the University of London and editor of the *British and Foreign Medical Review,* Carpenter wielded more influence over the curriculum and education of medical students in Britain during the 1840s and 1850s than perhaps any other individual.^1^ Physiological treatises were an essential genre that helped to establish authority in an era in which no firm grounding for medical truth existed, and they enjoyed a readership extending well beyond medical students and doctors (Porter 1992, p. 11; Digby 1994; Lane 2001, p. 23). Carpenter’s ideas had lasting popular appeal as well. Shannon Delorme has observed that Bram Stoker’s *Dracula*, published in 1897, includes terminology coined by Carpenter (2016, p. 5). Herbert Spencer cited Carpenter’s books as a key influence on his own thought and writing (Richards 1987, pp. 283–285), and Darwin was excited to have Carpenter’s support after publishing *Origin of Species.*^2^ The eldest son of the outspoken minister Lant Carpenter, William also wielded tremendous influence within the small but powerful Unitarian community, where his status as a physiologist was called upon to support the causes of temperance and abolition (Stange 1984). For medical students and lay readers alike, Carpenter’s views on the subject of heredity were authoritative, and his treatises represent an under-appreciated medium through which ideas about the laws of heredity took shape.

Anecdotal cases provided Carpenter with a means to argue that the inheritance of acquired characteristics was, in fact, beyond dispute: it was part of our everyday experience of the world, a familiar phenomenon that exhibited itself at work in agriculture, in the family, and in the geological record. As such, the inheritance of acquired characteristics provided a very powerful response to polygenists who argued against the unity of mankind, and it also provided a sound physiological basis for arguing the cause of the temperance movement and of education for the poor. These three causes formed the backbone of Unitarian church activities in the nineteenth century, and Carpenter’s family based its respectability and its social position on its contributions to these missions (Carpenter 1840; Delorme 2016; Desmond 1989, pp. 217–218; Schupf 1974; Stange 1984, pp. 125–131). While monogenists interested in arguing against slavery had been moving towards a theory of chance or accidental variation (Prichard 1813; Lawrence 1822; Wells 1971, p. 324), the moral value of education and of maintaining temperate habits could not be communicated via such a principle of heredity. This, I argue, explains why Carpenter abandoned the ideas of Prichard on chance variation in favor of an older base of evidence that would support the wider missions of the church.

By attending to Carpenter’s religious commitments, I also hope to show that we can resolve enduring questions concerning Carpenter’s attitude towards Jean-Baptiste Lamarck and the idea of transmutation. The inheritance of acquired characteristics is often referred to as “Lamarckism” within the historiography, and because William Carpenter’s early years find him in close proximity to Lamarckian sympathizers and proponents of transformist doctrines, it is important to distinguish Carpenter’s views on heredity from his views on Lamarck. Many historians uphold that there is a strong association between the inheritance of acquired characteristics with the influence of Lamarckian ideas in Britain (Bowler 2009, p. 86; Desmond 1989, p. 60; Zampieri 2009, p. 342; Galera 2017; Turbil 2017). “Lamarck was committed to progress,” as Michael Ruse puts it (1993, p. 53). The inheritance of acquired characteristics, according to this tradition, held political meaning, and was part of a larger cult of progress that took hold of British intellectuals in the nineteenth century. As I argue below, Carpenter rejected the ideas of Jean-Baptiste Lamarck in the 1840s and remained dedicated to progressive development as opposed to ideas of transmutation. The inheritance of acquired characteristics was a ubiquitous and widespread belief—animal breeders relied upon it, and the public viewed it as a familiar and reasonable assumption (Corsi 2011, pp. 15–16; Burkhardt 2013, p. 795; van Wyhe 2004, p. 193). In the case of mental illness, the inheritance of acquired characteristics was the dominant view of the nineteenth century (Waller 2002; Gausemeier 2015; Porter 2018). In the next section, I show that by looking to Carpenter’s religious commitments, we gain a clear perspective on how he separated the inheritance of acquired characteristics from “Lamarckism.” Following this, I explore the representation of heredity in three aspects of Carpenter’s work: his physiological treatises, his contributions to the temperance movement, and his original research on molluscs.

## Lamarckism and Unitarian Faith: Competing Ideologies in Early Carpenter’s Work

Carpenter encountered Lamarck’s ideas through his studies of invertebrates in medical school, and also through reading Charles Lyell. In these early years the influence of his father, Rev. Lant Carpenter, loomed large over his career. English Dissenters were eager, at the start of the nineteenth century, to “dissociate themselves from the charges of subversion in an age of war” (Adelman 1984, p. 67), and Unitarians were no exception. During the 1830s and the early 1840s, the very future of the Unitarian church was at stake. Due to their abandonment of belief in the Trinity, Unitarian ministers were outside mainstream belief and stood ready to lose control of property and institutions founded before 1813 (Andrews 2003, p. 5). It was only in 1844 that the Unitarian church was spared the effect of numerous legal cases by the passage of the Dissenters’ Chapels Bill (Watts 1995, pp. 547–548).While political battles threatened to remove the ownership of churches and educational institutions from Unitarian ministers in the 1840s, many in Britain perceived a threat of revolution within Unitarian theology (Andrews 2003; Corsi 1988, p. 57). Respectability was the chief means by which Unitarians contested these suspicions and attacks on the church. This often took the form of charity: establishing schools for the poor, promoting the abolitionist cause, and contributing to the temperance movement. Unitarian charity and missionary work stretched back to the eighteenth century, but from the 1840s it came to form an essential part of the respectability that membership in the church offered (Watts 1995, pp. 637–638). Unitarians won a number of political victories in the first half of the century, and philanthropic works played an important part in moving the church beyond the radical and negative connotations it held at the close of the eighteenth century (Watts 1998, p. 180).

Science and charity formed two routes towards respectability. Like many Unitarians of the time, the Carpenter family viewed scientific inquiry as contributing to and enriching the Christian faith, and they were skeptical and fearful of the materialist arguments that flourished elsewhere under the banner of the church (Corsi 1988, p. 57). William Carpenter’s decision to pursue a publishing career in science had clear ties to his commitment to the cause of abolition (Stange 1984; Desmond 1989; Delorme 2014; Winter 1998; Winter 2008). So too did it support his commitment to temperance and education reform. The 1840s was a period in which Unitarian preaching shifted to ally with “social elites,” and engagement with science remained part of this shift (Corsi 1988, p. 201). As William’s father, Lant Carpenter wrote: “There never was a profounder veneration for the Gospel, than exists at present among the highest in the walks of science” (1840, p. 5). As a physiologist, William Carpenter was in a position to provide a scientific account of the unity of mankind, a key theological justification for the abolition of slavery and the extension of Christian missions in the colonies. As he maintained: “There is no real disagreement between *true* Science and *rightly-interpreted* Revelation” (1845a, p. 246). Carpenter viewed religion as instrumental to the improvement of the race, and he used the principle of the transmission of acquired habits to justify this position. Faith remained a determining factor in his life. He was never impressed by Unitarian practice in London, complaining to his brother in 1847 that: “I think that Unitarians are pretty nearly as likely to be Pharisaical as Trinitarians, if placed in the same circumstances” (Carpenter 1889, p. 43).

Carpenter was intimately familiar with Lamarck’s work; indeed, Carpenter’s interest in foraminifera depended on Lamarck’s arguments against the systematists, discussed below. However, this familiarity was neither the source of Carpenter’s interest in the inheritance of acquired characteristics, nor did Carpenter associate the principle with the transformist ideas of Lamarck. Like many in the 1830s and 1840s, Carpenter viewed questions concerning heredity as founded on evidence, and questions concerning transmutation as speculative and philosophical. On the question of Lamarck’s transformist doctrine, Carpenter wrote that it “has been most deservedly ridiculed by almost every writer who has alluded to it” (1845b, p. 176). Carpenter also wrote to Ada Lovelace that the reasoning in Chambers’ *Vestiges* on the subject of transmutation was “loose and inconclusive,” suggesting that he did not hold a secret commitment to evolutionary ideas.^3^ In his review of *Vestiges,* he made clear what anecdotal evidence could and could not support:

The *known variability* of certain other species does not much help his doctrine; for though there are many breeds of dog and horses, sheep and oxen, they are all dogs and horses, sheep and oxen; differing in subordinate peculiarities, but in no instance becoming elevated towards any higher form, or acquiring any entirely new faculty. (Carpenter 1845b, p. 177)

In other words, heredity and transmutation were unrelated queries. The kinds of anecdotal evidence used to debate the laws of heredity were insufficient to debate transformism because no one had ever witnessed speciation. Lyell’s own *Principles of Geology* (1830–1833), a key source of information on Lamarck for the young Carpenter, rejected Lamarck but allowed that: “Some acquired peculiarities, of form, structure, and instinct, are transmissible to the offspring; but these consist of such qualities and attributes only as are intimately related to the natural wants and propensities of the species” (1832, p. 611). To support the idea, Lyell borrowed cases reported by John Corse to Joseph Banks in 1799 detailing the training of elephants in Bengal (Corsi 1978). While Corse made no mention of the inheritance of acquired characteristics, Lyell used his descriptions of elephants as an example of the hereditary effects of domestication—and, most importantly, of their limits. Elephants were naturally capable of domestication; Lyell viewed some of their domestic habits as the product of the inheritance of acquired characteristics. His concern was that there existed a natural limit to this progress. Carpenter repeated this exact principle. “Acquired peculiarities,” Carpenter cautioned his reader, “are seldom reproduced in the offspring, unless they have a relation with the natural habits and physical wants of the species” (1839, p. 421). Carpenter appreciated the careful position Lyell had adopted on Lamarck and the inheritance of acquired characteristics, and he replicated it perfectly in his earliest work.

Carpenter clearly viewed Lamarck’s approach as wrong—but he did not necessarily reject transmutation along with Lamarckism. Carpenter’s position on the question of transmutation in the 1840s has long been disputed in historiography, but no discussion of Carpenter’s views on the subject has ever referred to his writings for *The Inquirer,* where, writing to an audience of religious readers, Carpenter provided his clearest position on the question of transmutation prior to his reviews of Darwin’s *Origin. The Inquirer,* founded in London in 1842, styled itself as “useful to various classes of readers” and a “family newspaper” (1842, p. 1). Writing in 1845 on the harmony between science and religion, Carpenter felt there was little to choose between transmutation and separate acts of creation for the various species that populate the globe:

If we endeavour to trace back any race of Organized beings to its first origin, we find that we cannot assign to it any other commencement, than a certain union of the particles of Inorganic matter, which were then caused by the Creator to enter into new combinations, and to exhibit properties not previously manifested. Now whether each race had a *distinct* origin, or whether all had a *common* origin, the idea of *invariable plan,* or *law*, may be equally well entertained. It comes in the end to nearly the same thing. (1845a, p. 358)

Carpenter’s indifference to the question of transmutation might have concealed his strong preference for the fixity of species, but it also affirmed his belief that science could not dilute faith or threaten the power of God—it was present or absent, and there was no room within the debate for prevarication. “There is no half-way resting-place,” Carpenter warned, “between the opinion that matter is self-existent, and the idea that the operation of the Deity is *still* as constant and immediate as it ever has been” (1845b, p. 246).

Respectability, philanthropy, and science all worked together to advance the Carpenter family. Carpenter’s father, his brothers, and his sister were all deeply committed to abolition, temperance, and the education of the poor—but the role to be played by religion in this work was never questioned. When he left his father’s church in Bristol, this meant also “the loss of public worship” for Carpenter as a Unitarian (Carpenter 1889, p. 33). The stigma of Unitarian faith was surprisingly durable through the nineteenth century. During these tumultuous decades, William Carpenter’s father advocated for the abolition of slavery and education for the poor, establishing a girls’ charity school and an infant school in 1826 alone (Carpenter 1875, pp. 120, 166–167). Unitarians established a network of charity schools in Birmingham, Manchester, and Bristol, and they steadily grew the network through the first half of the nineteenth century (Watts 1998, pp. 170–172). But even as late as the 1850s, these schools faced exclusion from institutions like the Ragged School Union on theological grounds (Watts 1998, p. 175). Lady Byron, the wife of the poet Lord Byron and mother of Ada Lovelace, played a key role in funding these projects and lending respectability and institutional connections to the Carpenter family. William Carpenter was selected as the tutor for Ada Lovelace’s children and, some years later, Mary Carpenter was given the funds to establish the Red Lodge Reformatory. With the assistance of Matthew Davenport Hill, Mary continued to establish educational programs for the poor and also advocated for penal reform (Schupf 1974; Goldman 2004, p. 144). While most of the correspondence between William and Mary has been lost, a few preserved exchanges demonstrate that these campaigns for social reform were inextricably linked to the role played by religion in the future state. As William Carpenter wrote after the revolutions of 1848:

We can never forget this year. I cannot but believe that it is only the commencement of a more enlightened and progressive state, and that the demonstrations of popular force which it has exhibited will prevent for the future anything like a return to the arbitrary systems of the past. And one most hopeful sign has been that there has been nowhere any reaction against religion. (1889, p. 45)

Mary Carpenter viewed ragged schools as the necessary response to what she termed “moral disease” (Creese 2000, p. 358). Fitting into Daniel Pick’s (1989) wider discussion of degeneration theory in the nineteenth century, Mary Carpenter viewed juvenile delinquents as “patients” suffering from what she referred to as “the stamp of a deep-rooted and hereditary malady” (1853, p. 161). Within Unitarian theology, “moral disease” was commonly interchangeable with sin, to signify that as “sickness is not merely absence of health,” so too sin is not merely the absence of goodness (Stebbins 1874, p. 444). Mary, like many of her peers, viewed “moral disease” as relying upon the inheritance of acquired characteristics. To quote from a case she related in a later work:

A woman in deep grief came to ask information from me how to learn any particulars respecting her son, who had lately committed suicide in a convict prison. … She had made an unhappy marriage, and her husband, being too much given to drink, treated her cruelly. Before the birth of this poor young man her husband had been particularly brutal to her. The young child early showed signs of great irritability, and of a violent uncontrollable temper. (M. Carpenter 1864, p. 45)

Mary was heavily influenced by her brother’s work on the heritability of habits, particularly his views on the inheritance of “acquired habits of thought” (M. Carpenter 1853, p. 384). In her work *Juvenile Delinquents* (1853), she included an excerpt from her brother’s work on mental habits and physiology (pp. 383–388). In this way, Mary Carpenter not only drew upon her brother’s work to justify and frame Unitarian mission work with the poor, but also introduced William’s physiological works to a wider readership. William Carpenter’s work came to be increasingly important, not only to the work of his sister, but also in establishing a physiological basis for the ideals of education, temperance, and abolition that were key to the churchs’ wider social roles.

## Anecdotal Evidence and Compilation: Working Towards a Principle of Heredity

So far, I have considered Carpenter’s early attitudes towards Lamarck and his involvement in the Unitarian church. In this section, I return to 1839 to trace how Carpenter compiled the anecdotes that he used to support his views on heredity. Carpenter may have rejected the transmutationist ideas of Lamarck, but he still believed that “the philosophic naturalist” was responsible for “investigating the degree of variation which each is liable to undergo” (Carpenter 1839, p. 414). This job description stemmed from his own anxieties that Christianity was under threat from scientific efforts to prune non-European races from the human family tree. From the 1830s onwards, Britain experienced a profound cultural shift in its attitudes towards mixed race marriages (Salesa 2011, p. 133). While Prichard had been the dominant authority on questions of the human races and intermarriage, at the time of his death his views were being eclipsed by the American polygenists (Augstein 1998; Kidd 2006; Keel 2013; Salesa 2011). For Carpenter (and many other Unitarians), the historical accuracy of Genesis had already been abandoned: what mattered instead was what Kidd has described as “the central theological truth of mankind’s unitary origins” (2006, p. 130). Non-European races were certainly intellectually inferior in Carpenter’s judgement, but this inequality stemmed from the absence of European habits of study and the influence of Christian civilization. He maintained that:

whilst the American and Oceanic races appear doomed to extinction as pure races, wherever they come into contact with Europeans, there is no evidence that such is the case with those of Mongolian or of African descent; the latter, indeed, hold their ground with remarkable tenacity, and we may not improbably regard them as destined, under the influence of Christian civilization, to bear an important part in the future history of Mankind. (Carpenter 1849, p. 1365)

The inclusion of all races into the Christian destiny was always of central importance to Carpenter, and the inheritance of acquired characteristics offered not only as a means of explaining the existing variations between races, but also of the power of Christian education to reform and improve. The causes of temperance and education, as I show below, became increasingly important to him through the 1840s, as he began to publish on the subjects of temperance and mental physiology.

It is important to consider the criticisms that were directed towards the inheritance of acquired characteristics by Carpenter’s contemporaries. In 1839, the young Scottish anatomist Allen Thomson, a classmate of Carpenter’s, compiled a list of 41 common stories repeated by farmers and the public, and cautioned: “We look upon all these alleged facts with distrust. Many of them are coincidences; others, we suspect, are false” (1839, p. 472). Cases of cats born without tails received mention, as did the instincts of pointers and hunting dogs. The educability of horses and the wariness of foxes that lived in areas where hunting is practiced—all such stories, Thomson claimed, were misleading and false. Twenty-five years earlier, James Cowles Prichard’s *Researches into the Physical History of Mankind*, first published in 1813, contained a refutation of the inheritance of acquired characteristics (p. 195). The evidence for the idea was, in his eyes, scant, and by no means strong enough for the task of confronting polygenist arguments against the old and familiar arguments that adaptation to climate had produced the different races. When criticizing the inheritance of acquired characteristics, Prichard commented that the stories in its favor seemed suspect:

The authors who have brought such examples as these in defence of their opinions, would not probably have thought them worth recording, or indeed deserving of the smallest notice, if they had not happened to coincide with the systems they were advocating. (1813, p. 197)

Prichard understood chance variation to produce races and varieties (1813, p. 26). As Augstein (1998) has shown, these ideas fit Prichard’s Unitarian faith. His aim was merely to reject a body of evidence that he viewed as specious and conflicting with his views on the moral responsibility of the individual. The inheritance of acquired characteristics, in contrast, described sin as arising in the choices and actions of one’s ancestors. Based on Carpenter’s friendship and admiration for Prichard, it seems difficult to maintain that he would champion the inheritance of acquired characteristics merely on the grounds of abolitionism.

The cases and examples Carpenter compiled all evidenced one idea: civilization and cultivation prompt individuals (plants, animals, and humans) to alter their habits. These alterations can take the form of behaviors or physical traits, but they will—when they align with the natural propensities of the species—be transmitted to offspring. In his *Principles of Comparative Physiology* (1839), Carpenter introduced the key cases and examples that he would continue to advance as late as his paper on the inheritance of acquired characteristics in 1873. From the plant realm, Carpenter cited the ability of gardeners to convert single flowers into doubles by the power of cultivation—a familiar argument that cultivation could induce transmittable new habits into varieties of plants (1839, p. 419). Cultivation was a useful source of evidence, and like many others, Carpenter appealed to the authority of nurserymen and plant breeders. He attended to the practices at his local plant nursery in Bristol, and he reported in the first edition of his *Principles* that the nurseryman at Durdham Down had demonstrated that three Orchideous plants previously regarded as belonging to distinct genera could be produced from the same parent, if cultivated properly (1839, p. 415). Such examples had immediate significance for debates over the origin of the human races, despite their apparent remove. A couple of years after the second edition of the *Principles* was released, Josiah Nott delivered a series of lectures in which he likened the “retrograde movement” of freed Africans towards barbarity to that of poorly cultivated plants (1844, pp. 40–50). In his influential work on the *Varieties of Mankind* (1849), Carpenter continued to appeal to plants to support the belief that gardeners and plant breeders were well aware of the power of cultivation to establish new varieties. He appealed to the familiar work of the botanist William Herbert, who was able to raise primrose, polyanthus, cowslip, and oxlip, all cultivated varieties, from the seeds of the same plant (Carpenter 1849b, p. 1305; Herbert 1837). While Herbert believed in the inheritance of acquired characteristics, not all plant breeders and gardeners regarded these traits as *acquired* by any means—the idea of “sports” and chance variations were important to these knowledge communities. But Carpenter represented these narratives to fit his own view that the influence of cultivation was the causal factor in the appearance of these hereditary traits.

Carpenter was also willing to break with the views of Prichard and to attach authority to an older canon of stories about the inheritance of mutilations. In addition to a discussion of “cats deprived of their tails,” Carpenter claimed in his *Principles* that: “instances are on record in which dogs, that have been deprived of their tails by accident or design, have produced puppies with a similar deficiency” (1839, p. 422). The “record” mentioned was the agricultural and sporting press, where such anecdotal cases abounded. To quote an example from *The American Turf Register:*

Accidental injuries sometimes become hereditary blemishes. Little Billy had one eye put out when a colt, by accident, the other remained good during the whole time he was on the turf; after he became a stallion, he lost his other eye by a stroke of the whip from his groom, in a few years after, many of his stock were blind, and this did not occur until he had been some years in that condition. (Anonymous 1832, p. 74)

The emphasis from Carpenter, once more, was that such cases and stories testified to the ubiquity of the phenomenon—for animal breeders and farmers, such cases fit their ordinary and practical expectations of breeding. They conflicted with Carpenter’s contention that only traits allied to the natural propensities of the species were transmitted, but Carpenter clearly valued them as a base of familiar evidence too much to discount them on such grounds. He would make such a departure again in the case of alcoholism (considered below).

Like many others, Carpenter was convinced that a vast and useful experiment on heredity was playing out in the colonies, where the introduction of European breeds and varieties brought about an opportunity to observe the hereditary effects of new climate and soil (Desmond 1989, p. 301; Müller-Wille and Rheinberger 2012). A well-respected collection of these anecdotal cases had already been investigated and compiled by the French physician and travel writer François Désiré Roulin (Osborne 1994). Roulin communicated a highly influential report on climate and heredity in 1828 to the *Academie des sciences*, which later confirmed for Carpenter that the hereditary transmission of acquired habits was now a familiar phenomenon, widely observed in farms and colonies abroad. Of particular interest to Carpenter (and also Lyell) was an account of how dogs imported to Colombia had adopted new hunting instincts:

In a mongrel race of dogs employed by the inhabitants of the banks of the Magdalena almost exclusively in hunting the white-lipped Pecari, a peculiar instinct appears to have become hereditary, like that of the pointers and other dogs of this country. The address of these dogs consists in restraining their ardour, and attaching themselves to no animal in particular, but keeping the whole herd in check. Now among these dogs some are found which, the very first time they are taken to the woods, are acquainted with this mode of attack; whereas, a dog of another breed starts forward at once, is surrounded by the Pecari, and, whatever may be his strength, is destroyed in a moment. (Carpenter 1839, pp. 421–22; Lyell 1832, pp. 39–40; Roulin 1829)

The wording is largely based on a translation of Roulin’s article published in the *Edinburgh Monthly* in 1829, which appears verbatim in Lyell’s *Principles of Geology* (1832). Roulin’s testimony was trustworthy, not only because of his status as a physician, but also because his observations had been communicated to the *Academy*, where Geoffrey Saint-Hilaire quickly endorsed and elaborated upon Roulin’s cautious ideas as to how such habits became hereditary (Appel 1987, p. 133). Roulin’s accounts of such cases from the colonies fit other sources that Carpenter employed. He frequently made mention of an anecdote from Thomas Bell about how domestication of two Australian dingoes had yielded, when bred, puppies with spots, when their parents had been uniformly brown (Carpenter 1849b, p. 1305; Bell 1834, p. 203). Such cases helped to illustrate the effects of cultivation. But Carpenter was also interested in travel observations that confirmed the effects of climate on such traits—even if they included notions that some climates (for whatever reason) tend to shape different species in the same manner. “It is a curious and very significant fact,” he explains, “that the sheep of the Cape of Good Hope, which are descended from the European stocks, should exhibit the same tendency to the accumulation of fat about the rump, as is seen in the human races indigenous to that region” (Carpenter 1849b, p. 1312). Carpenter was referring to Angola sheep, gaining his opinion from reading the veterinarian William Youatt’s discussion of their unusual features (1837, pp. 119–120).

Alongside these animal and plant anecdotes, Carpenter discussed cases from ethnology and medicine. Carpenter was particularly influenced by the controversial work of the London physician Alexander Walker, whose book *Intermarriage* (1838) included numerous examples and cases of the benefits of crossing races and varieties to the quality of the human stock. Walker had included an example from John Hancock, doctor and zoologist whose travels in South America produced communications that frequently provided material for the periodicals and popular encyclopedias of the 1830s. Walker had instigated a correspondence with John Hancock from which he gained new stories and material for his *Intermarriage*, among which was the claim by Hancock that he had witnessed “whitening” among slaves and African servants in the Americas and Europe due to the effects of climate (Walker 1838, pp. 275–278; Carpenter 1841). The obvious failure of such a principle to account for the variety of skin tones in tropical and temperate climates had been debated since the Enlightenment, making it all the more surprising that Carpenter would endorse it (Livingstone 2008 p. 59; Gould 2006). Responding to these convincing arguments, Prichard himself argued in 1813 that “white races of people migrating to a hot climate, do preserve their native complexion unchanged, and have so preserved it in all the examples of such migration which we know to have happened” (p. 231). But Carpenter’s principle of heredity, as we saw in the case of animals, placed a tremendous emphasis on the power of climate and circumstances to alter the physical appearance of species:

among some savage nations of North America and New Holland, precisely the same notion of direction is manifested, as is evinced, in a degree scarcely more remarkable, by the lower animals; individuals traversing pathless forests for the first time without swerving in the least from the direct line towards the point at which they are aiming. (Carpenter 1839, p. 423)

Carpenter may have found such stories in the writings of Humboldt, whose descriptions of American Indians invoked hereditary powers (Walls 2009, pp. 58–64), or he may have encountered them in Alexander Walker’s *Intermarriage* (1838), which had included some excerpts from correspondence Walker claimed to receive from Thomas Andrew Knight, who had recently published a collection of cases supporting the inheritance of acquired traits (Knight 1837). In these passages, Knight is quoted as having written to Walker that: “The offspring of a family of American or Australian savages, would more readily acquire the power of tracing the steps of an animal in a trackless forest, than the child of an educated English family would do” (Walker 1838, p. 179).

Carpenter used his treatises as a space in which to gather cases and examples that could respond to the growing threat of polygenism. While monogenists that he admired (like Prichard) had rejected these stories, Carpenter determined that the movement should change tack and appeal to the idea that cases like these were illustrative of experiences familiar to all. When we consider Carpenter’s interest in temperance and education, his preference for the inheritance of acquired characteristics becomes clearer.

## Temperance

If Carpenter’s commitments to the abolition of slavery and the temperance movement provided key motivation for his views on heredity, the temperance movement was by no means diminished in moral significance compared to the cause of ending slavery. Russell Lant Carpenter (William’s brother) maintained that intemperance was worse than slavery, a position that reflects the wider imbalance of church activity in favor of the temperance cause (Stange 1984, p. 35). Whereas there are many accounts of heredity that could be enlisted to argue for the unity of mankind, the temperance movement was inextricably tied to questions about the effects of acquired habits—particularly the acquisition of intemperate habits on subsequent offspring. As one minister explained in a temperance sermon from 1841: “Now it has come under our personal cognizance, that children do sometimes inherit their parents’ fondness for strong drink, and, very generally, their parents’ diseases” (Antliff 1841, p. 239). However, such views were by no means uniform. In the *Boston Medical Journal*, a physician, skeptical of the over-reliance on acquired characteristics, wrote in 1835: “Most children who are brought up with their parents, easily contract their habits. Often the drunkard’s or the libertine’s child should accuse the parent less for transmitting gout and enervation, than for setting examples of the vices from which these effects result” (Anonymous 1835, p. 368). Thus, while there were voices insisting within the wider medical realm that it was beyond question that acquired habits informed the constitutions of offspring, there remained doubt and skepticism over whether or not such explanations were necessary for understanding the nature of nervous disorders (as intemperance was then viewed). Carpenter did face a choice over whether or not to advocate a hereditary perspective on intemperance, but chose to focus exclusively on anecdotal and statistical evidence that supported the hereditary view.

In 1849, John Forbes, the former editor of the *British and Foreign Medical Review* and court physician to Prince Albert, announced a prize essay worth 100 guineas on the subject of the physiological effects of alcohol consumption (Carpenter Carpenter 1849a, pp. i–iv). Such competitions were part of the early strategies of the temperance movement, and they extended to all educational levels. Carpenter’s essay was to be the most influential and celebrated, but *The National Temperance Society* also ran essay competitions for working men and published the winning essays (Couling 1860, p. 201). Prize essays were a key genre within the temperance movement, and (as mentioned above) Carpenter’s essay would earn him significant popularity and respect within the temperance movement in Britain and America, where his work was regularly reprinted within the temperance press. He sought to rally evidence from asylums, phrenological magazines, and medical publications to support the view that the habit of drinking, once acquired, has hereditary consequences which necessitate the total elimination of alcohol from the diet:

Sad experience has shown, that a large proportion of mankind *cannot*, partly for want of the self-restraint which proceeds from moral and religious culture, be temperate in the use of alcoholic liquors; and that the reformation of those who have acquired habits of intemperance *cannot* be accomplished by any means short of entire abstinence. (Carpenter 1851a, p. xix)

Carpenter never sought to clarify how such a view fit within the restriction that acquired habits can only become hereditary when they fit the natural propensities and needs of the species. But he had a wealth of evidence that he could borrow from the asylum reports published in Britain and America, which were at this time producing volumes of statistical insights into the hereditary causes of insanity. “There is an Asylum in the East of London,” Carpenter wrote, “where the proportion of cases attributed to intemperance alone amounted to 41.07 per cent” (1851a, p. 35). From Samuel Gridley Howe, one of several influential American asylum doctors that linked idiocy to moral crimes (McDonagh 2008, p. 262), Carpenter borrowed a table purporting to show that out of a sample of 300 idiots, 145 were the offspring of habitual drunkards (Carpenter 1849a, p. 43). Carpenter’s use of the table made it a handy, oft-quoted fact that would circulate for decades until, as McDonagh observes, Henry Maudsley dismissed it as unfounded in 1867 (McDonagh 2008, p. 264). The table is interesting because, despite Carpenter’s insistence that the hereditary nature of intemperance followed the pattern of scrofula or gout, it clearly is importantly different. Parents with tubercular constitutions will have children also prone to tuberculosis. Parents with gout will have children prone to gout. But Carpenter, in 1849, affirms that asylums in Britain and America have adequately demonstrated that intemperate parents can give birth to children suffering not only from idiocy, but also from epilepsy, mania, monomania, melancholia, and so on. “There can be no doubt,” Carpenter argued, “that those who have weakened and disordered the nutrition of the brain by habitual Intemperance, are far more liable than others to be strongly affected by those causes, moral or physical, to which the Mental Derangement is more immediately attributable” (1849a, p. 31).

Carpenter speculated that in many of the cases labelled “hereditary” in the published statistical tables from asylums, alcohol had been an exciting cause—and he viewed this as further likely when he considered that pauper asylums contained many more intemperate patients than did those that catered to the higher classes (1849a, pp. 44–45). But in order to argue that intemperance led to hereditary insanity, he needed evidence, and so Carpenter borrowed heavily from *The Phrenological Journal* and the asylum reports of William Hutcheson, superintendent of Glasgow Asylum and head of the local phrenological committee in that city.^4^ While Carpenter remained dubious and skeptical about the tenets of phrenology, his views on human heredity were ultimately built on the evidence that filled the pages of the Phrenological Association’s journal. As Delorme (2014, p. 59) observed, Carpenter began his career in Edinburgh by entering the debate over phrenology as a skeptic; yet he did not do so without writing to Combe to express just how much he had personally learned and benefitted from phrenological literature. Carpenter had read Browne’s essays on hereditary insanity and was struck by the asylum surgeon’s observation of intemperance and the family. He was particularly interested in Browne’s observation that three of his patients at Dumfries in 1841 owed their “unhealthy action of the brain” and idiocy to intemperate parents (Carpenter 1849a, p. 52). Carpenter used *The Phrenological Journal* as a source for his anecdotes and medical cases, from which he drew a similarly vague case concerning two parents that were only mildly intemperate, and yet bore a child that was “completely idiotic” (1849a, p. 53).

The cause of temperance, for Carpenter, required institutional intervention—the hereditary consequences of intemperance providing a key motivation for such a view. In this, William’s position was allied with that of his sister Mary, who upheld in the cause of reformatory schools that “there is one stamp of degradation on them all [the perishing classes], which must make them an hereditary burden to the country” (M. Carpenter 1851, p. 72). The solutions to these hereditary dangers took the form of institutions and charities run by Unitarian churches—or, in many cases, by initiatives of William Carpenter’s siblings. His views fit into a wider narrative already identified in Unitarian theology during the 1850s, in which natural laws could be identified as producing the social conditions the church sought to address (Corsi 1988, p. 201). In other words, Carpenter’s position on the inheritance of the acquired effects of intemperance fit a wider shift in the Unitarian church in which the physiological authority that Carpenter could bring to such questions spoke to a congregation that was wealthier and more powerful than it had been in the early 1800s.

## Carpenter’s Belief in the Inheritance of Acquired Characteristics and His Observational Work

So far, I have argued that the choices Carpenter made in selecting varieties of evidence in debates over heredity demonstrate the real influence that his commitments to abolitionism and the temperance movement held over his views. While Carpenter’s work on the physiology of invertebrates is obviously removed from these concerns, he nonetheless used this work as a potential pool for future evidence on the mechanisms of heredity. He explicitly maintained that this work provided a further body of evidence in support of his beliefs about the inheritance of acquired characteristics, despite the very practical difficulties in maintaining such a position. Carpenter’s original research focused on the study of foraminifera. His family connections to the manufacturers of lenses and microscopes, and his apprenticeship to the eye surgeon John Bishop Estlin, both provided a familiarity with the technology involved, while his education under Robert Grant in London included an introduction to Lamarck’s taxonomy and work on molluscs (Delorme 2016; Desmond 1989). While Carpenter clearly valued anecdotal evidence, this was by no means because he lacked skill and expertise in technical observation. In 1843, Carpenter’s microscope could make out details at 1/6000th of an inch, allowing him to perceive the structure of the minute tubes that composed the structure of shells and to put forward new classifications based on structural similarities alone:

I cannot but think that the value of the microscope as an instrument of geological research, must be at once evident from these statements. The genera *Terebatula*, *Spirifer*, and *Producta* may be at once distinguished from each other, and from all other shells, by the characters supplied by a fragment of shell, which a pin’s head would cover. (Carpenter 1843, p. 384)

It is important to weigh Carpenter’s dedication to anecdotal evidence in respect to his belief that microscopic analysis could revolutionize the understanding of variation between mollusc specimens (and later, foraminifera). Carpenter rejected the transmutationist views espoused by Lamarck, but Carpenter *was* in alignment with Lamarck’s position against the systematists, and he argued that microscopic study of the anatomy of shells and the skeletal remains of Echinodermata could anticipate conclusions reached through study of anatomy (1843, p. 386). Technology was also improving the means of accessing specimens from the seabed, with the first steam-powered bucket dredger put to use in 1798 (Eisma 2005, p. 105). In the 1840s, steam bucket dredgers grew increasingly common within coastal port areas around the world, providing greater quantities of specimens. Data was abundant and cheap, and Carpenter hoped to use it to provide a new evidence base for debates over heredity. Specimens like these provided an accessible and inexpensive base of evidence upon which to develop original work. At the end of his papers, Carpenter included a note requesting submissions of shells from readers and collectors (1843, pp. 377–390). He was by no means beholden to anecdotal evidence because he lacked the technical skills to inquire into the laws of heredity by other methods and means.

There is no reference in Carpenter’s original research articles to accidental or spontaneous variation (see Carpenter 1843, 1849a, b, 1856). Having received a grant to collect and examine the structure of mollusc shells, Carpenter admitted that variations are observable: “The size of the elementary parts is the chief point of difference” (1843, p. 2). Yet he did not speak to the significance of these variations, writing only: “I am not yet prepared to speak with certainty” when addressing the variations among his specimens (Carpenter 1843, p. 2). Carpenter was impressed by a paper published by the Royal Society in 1833 that attested to have discovered a harbor in Plymouth where specimens of *Littorena petroea* showed divergent features whether they lived within the conditions of the harbor or outside of it (Gray 1833; Carpenter 1839). Simple organisms were important to the question of variation because Carpenter (and many others) believed that the simpler an organism was in organization, the more susceptible it was to variation (Carpenter 1839, p. 419). But what was the cause of this variation? Was it accidental or spontaneous, or was it caused by external circumstances?

Darwin was studying barnacles at the same time and was struck by what he referred to as “the variability of every part.”^5^ Variation in individual barnacles greatly frustrated Darwin’s efforts to classify them and provided strong evidence for the idea of spontaneous variation (Love 2002, p. 252; Stott 2003; Richmond 1989; Southward 1983, p. 69). But Carpenter, from his early publications in the 1840s, was unwilling to conclude that variation among his specimens had no external cause. “That peculiarities of structure sometimes arise independently of external agencies can scarcely be doubted.” (Carpenter 1839, p. 421). He termed this “accidental variation.” Carpenter distinguished such variation from the inheritance of “acquired peculiarities” by stressing that the latter must bear a relationship to the natural habits and climate of the species (1839, p. 421). But Carpenter’s views were conflicted, and after 1841 he always maintained that: “The simpler the condition of any organism, the more susceptible it is of being modified in form and structure by causes external to itself” (1841, p. 169). By 1849, Carpenter had assembled over 600 slides that formed his evidence base; as his access to foraminifera from dredging across the empire improved, he increased the size of this collection to over 2000 slides (Murray and Taplin 1984, pp. 1–2). Deep-sea soundings were producing samples from colder, darker seabeds than before, allowing Carpenter to enlist climate and temperature as causes of variation (1862, p. x). In 1849, based on his collection of nummulite fossils, Carpenter suggested that the variations in size and shape that these unicellular organisms took might be caused by variations in the abundance of calcium in the prehistoric environment (1849b, p. 30). The reasons for his insistence on this point became clear in 1862, when he finally published his large work on foraminifera.

In his *Introduction to the Study of Foraminifera* (1862), the culmination of his studies, Carpenter continued to caution that the accidental variations observed in foraminifera must be attributed to climate, noting that the variations in their shells were attributable to “the influence of some external condition, probably an excess in the proportion of carbonate of lime in the waters inhabited by these particular specimens” (Carpenter 1862, p. 122). Even monstrosities were, Carpenter believed, linked to climate:

Among some hundreds of specimens which I have examined from the coast of Australia, I have only met with five or six; among those yielded abundantly by the sand of the shore at Suez, such monstrosities are far more frequent, and the excess more pronounced … I am disposed, therefore, to regard them rather as resulting from the influence of external conditions, than as accidental varieties hereditarily propagated. (Carpenter 1862, p. 124)

Carpenter sought to use foraminifera to show that climate and external circumstances determine the variations we observe. Monstrosities—by definition cases of accidental variation—could also be explained (Carpenter insisted) by appeal to climate. His position remained unchanged from that put forward in his Fullerian geology lectures delivered in 1847, which state that climate and “conditions of existence” are always the forces at work in causing variation:

We seem entitled to conclude further, that there was formerly less diversity than at present prevails in these conditions of existence—such as temperature, food, character of the sea bottom, ocean currents, etc—which so remarkably modify the distribution of the existing species of this class, and, which seem, indeed, to be (if I may so speak) the very source of their variety. (Carpenter 1847–1848, p. 16)

Carpenter’s observational research on marine life always had political and religious significance, despite its apparent remove from such concerns. His introduction explains that the variety found within foraminifera is more abundant than the variety found in the human family, and yet it can be shown that foraminifera share a common descent (Carpenter 1862, p. ix). “This case,” Carpenter wrote, “is very analogous to that of the relationship between the various members of the family of Mankind” (1862, p. ix). As he explained: “The expectation of finding in the group of Foraminifera an entirely new and valuable body of material for the prosecution of this inquiry [common descent], was one of my chief reasons for applying myself to the systematic study of it” (1862, p. 13).

Despite the bewildering variations he observed, these years of research into foraminifera (roughly 1850–1862) did not lead Carpenter to endorse spontaneous or chance variation. On the contrary, he maintained the position he set out in 1853 that all appearance of “chance” or “spontaneity” is borne out of our limited understanding. Carpenter thought that what appears to our limited impression as chance and random was in fact the product of the environment. “There is no effect without a cause, and as the widest differences of this kind present themselves in those races which are most obviously amenable to the influence of external conditions, we seem justified in attributing to them agencies operating unostensibly upon the parents” (1853, p. 1038). Carpenter remained wholly resistant to the suggestion that there could be internal determinants of change, disconnected from external conditions. But such a theory of heredity was not supported by narratives and anecdotes: Carpenter relied almost exclusively on the ability of stories (and sometimes statistics) to trace characteristics and qualities from one generation to the next. Chance variation and sports could not be observed by following the narrative conventions that Carpenter discovered within the kinds of reported cases from gardening, agriculture, medicine, and travel writing.

## Conclusion

By the end of Carpenter’s life, when a divergence in the science of heredity was taking place, figures like Francis Galton (and later Bateson) were focusing on investigating variation. Yet Carpenter increasingly maintained that our everyday, familiar experience of heredity—the kind produced by animal breeders, plant breeders, and physicians that worked with families—was true and valuable, and ought to guide investigation (Carpenter 1873). In this article, I have argued that Carpenter used his own status and position to argue for the inheritance of acquired characteristics because the idea lent essential scientific support to the missionary work of the Unitarian church. In doing so, Carpenter helped to establish a divergent scientific approach to heredity that placed the principle of “like produces like” at its center, influencing the temperance movement, eugenics, and political economists like Herbert Spencer for decades to come.

As I have argued, the insistence on the primacy of the inheritance of acquired characteristics in Carpenter’s work has clear ties to his commitments to the abolition of slavery and the temperance movement, the two most important social movements of early-nineteenth-century Unitarianism. Further, the varieties of evidence that Carpenter preferred to employ— anecdotal cases borrowed from gardening magazines, agricultural publications, and phrenological magazines—testify to his conviction that the laws of heredity could be directly observed, and that by compiling cases, what was already known and familiar to his readership could be demonstrated just by a process of accumulation. In a sense, Carpenter never really put forward a theory of heredity at all. Rather, on the subject of heredity, Carpenter’s treatises argued that our intuitive, lived experience already contained evidence enough to confirm the transmission of acquired characteristics. The scientific press provided a means of demonstrating the universality of this experience. Articulating a scientific perspective on heredity consisted of assuring his readership that their experiences of the inheritance of acquired traits and characteristics were real and shared by others. This simplicity and emphasis upon the reader’s own knowledge and familiarity of the laws of heredity in action was continuous with other forms of knowledge that Carpenter hoped to elicit from his reader. Carpenter’s (presumed) reader already knew that the races of mankind were all potential converts to Christianity, and so too did the reader already know that vices like intemperance are transmitted from parents to offspring. The stories selected by Carpenter are occasionally unusual, but they are still selected for their familiarity. The laws of heredity, for Carpenter, are clearly visible in tracing the transmission of habits and illnesses within one generation of an intemperate family; so too are these same laws writ large within the cultivating practices that shape Britain’s gardens. Within the exotic examples Carpenter borrowed from travel writing, the influence of cultivation and civilization is a familiar and expected cause of the complex changes in coloration, shape, and behavior that Carpenter feels have been witnessed by authorities abroad.

As the inheritance of acquired characteristics gained prominence through the latter half of the nineteenth century, it came to be associated (to Carpenter’s chagrin) with the philosophical system of Herbert Spencer and also the “neo-Lamarckian” alternatives to natural selection. Carpenter was certainly aware of Lamarck’s ideas, but there is no evidence to support the suggestion that he saw any connection between the mechanisms Lamarck proposed to explain the transformation of species and the inheritance of acquired characteristics. The latter idea was presented by Carpenter (and others) as an idea that was already familiar to everyone, universally validated by experience. This does not provide evidence that the idea really was universal in the early nineteenth century. We cannot know with certainty what Carpenter’s or Combe’s readers believed about heredity before reading their works, if they believed anything concrete at all. More compellingly, this article demonstrates that a number of writers in Britain during this period adopted the same strategy in *telling* their readers that such an idea was universally upheld and vindicated by popular experience. Thus, a crucial aspect of the contest over the laws of heredity did not concern the results of experimentation or the opinions of elite scientific figures. Rather, the principal figures in the debate appealed to the public’s experience, and it was the construction of this court of authority that would ensure that the idea earned the same epistemological status as our knowledge that the sky is blue, and that an object at rest remains at rest.

## Footnotes

1 John Churchill’s publishing records show that he produced 1000–1250 copies with each edition of Carpenter’s various treatises and manuals, publishing eight revised editions of his Comparative Physiology and his Human Physiology between 1839 and 1854. (Documentation of the print runs are preserved in the papers of John Churchill, held at the University of Reading. For the editions cited, see: MS1393/M5 536, 580, 581; MS1393/M6 3, 329*, 211–212, 409; MS1393/M7 59–60, 281–282, 337–338; MS1393/M8/3.)* His treatise on the microscope (published in 1856 and 1857) saw 5000 copies printed—one of the highest in Churchill's records (MS1393/M8/201–202, 307). Carpenter was, with the exception of Robert Chambers’s anonymous *Vestiges*, arguably Churchill's best-selling author in the 1840s and 1850s—and Carpenter was heavily involved in the editorial revisions of the scientific content of *Vestiges* (Secord 2003).

2 Charles Darwin to William Carpenter, 3 December [1859], Darwin Correspondence Project, “Letter no. 2568,” accessed on 24 May 2019, http://www.darwinproject.ac.uk/DCP-LETT-2568. Also published in *The Correspondence of Charles Darwin*, vol. 7.

3 Papers of Ada Lovelace, Dep Lovelace-Byron, 169/222–223, Bodleian Library, University of Oxford.

4 Evidence of Hutcheson’s position within the Phrenological Society can be found in the “List of Members of the Phrenological Association, at 19th September 1840,” *The Phrenological Journal,* 13 (1841), 119–123. See also (Hutcheson 1842).

5 To J. D. Hooker, 13 June [1850], Darwin Correspondence Project, “Letter no. 1339,” accessed on 24 May 2019, http://www.darwinproject.ac.uk/DCP-LETT-1339. Also published in *The Correspondence of Charles Darwin*, vol. 4.
